# The Mitochondrial *m*-AAA Protease Prevents Demyelination and Hair Greying

**DOI:** 10.1371/journal.pgen.1006463

**Published:** 2016-12-02

**Authors:** Shuaiyu Wang, Julie Jacquemyn, Sara Murru, Paola Martinelli, Esther Barth, Thomas Langer, Carien M. Niessen, Elena I. Rugarli

**Affiliations:** 1 Institute for Genetics, University of Cologne, Cologne, Germany; 2 Cologne Excellence Cluster on Cellular Stress Responses in Aging-Associated Diseases (CECAD), University of Cologne, Cologne, Germany; 3 Center for Molecular Medicine (CMMC), University of Cologne, Cologne, Germany; 4 Department of Dermatology, University of Cologne, Cologne, Germany; Max Planck Institute for Biology of Ageing, GERMANY

## Abstract

The *m*-AAA protease preserves proteostasis of the inner mitochondrial membrane. It ensures a functional respiratory chain, by controlling the turnover of respiratory complex subunits and allowing mitochondrial translation, but other functions in mitochondria are conceivable. Mutations in genes encoding subunits of the *m*-AAA protease have been linked to various neurodegenerative diseases in humans, such as hereditary spastic paraplegia and spinocerebellar ataxia. While essential functions of the *m*-AAA protease for neuronal survival have been established, its role in adult glial cells remains enigmatic. Here, we show that deletion of the highly expressed subunit AFG3L2 in mature mouse oligodendrocytes provokes early-on mitochondrial fragmentation and swelling, as previously shown in neurons, but causes only late-onset motor defects and myelin abnormalities. In contrast, total ablation of the *m*-AAA protease, by deleting both *Afg3l2* and its paralogue *Afg3l1*, triggers progressive motor dysfunction and demyelination, owing to rapid oligodendrocyte cell death. Surprisingly, the mice showed premature hair greying, caused by progressive loss of melanoblasts that share a common developmental origin with Schwann cells and are targeted in our experiments. Thus, while both neurons and glial cells are dependant on the *m*-AAA protease for survival *in vivo*, complete ablation of the complex is necessary to trigger death of oligodendrocytes, hinting to cell-autonomous thresholds of vulnerability to *m*-AAA protease deficiency.

## Author Summary

Oligodendrocytes are cells of the central nervous system that produce the myelin sheath. Myelin production is extremely costly from the energetic point of view, and oligodendrocytes that are synthesizing myelin are particularly susceptible to mitochondrial dysfunction. However, the function of mitochondria in mature oligodendrocytes, after myelination is completed, has been poorly explored using genetic models. Here, we have generated and characterized mouse models expressing different levels of the *m*-AAA protease, a proteolytic complex preserving proteostasis of the inner mitochondrial membrane and respiratory activity. We show that oligodendrocytes are capable to cope with reduced levels of the complex, but undergo rapid death upon complete ablation of the *m*-AAA protease. Thus, the *m*-AAA protease is essential for cell survival, but oligodendrocytes are less vulnerable than neurons to a deficiency of the complex.

## Introduction

Oligodendrocytes are glial cells of the central nervous system (CNS) that produce myelin to enhance conduction velocity. Oligodendrocytes utilize high amount of energy to synthesize proteins and lipids to build up myelin [1] and depend on mitochondrial respiration heavily during differentiation and myelination, when they are dramatically susceptible to ischemia, energy deprivation, and oxidative stress [2, 3]. It has been hypothesized that post-myelination oligodendrocytes can undergo a metabolic switch to glycolysis, and provide metabolic support to axons, by supplying lactate as an energy source [2, 4]. In support of this hypothesis, oligodendrocyte-specific deletion of an essential assembly factor for complex IV, Cox10, did not lead to axonal degeneration or demyelination [2], strongly suggesting that these cells can survive a respiratory chain deficiency. Moreover, recent data suggest that oligodendrocyte mitochondria may be involved in specialized functions relevant for myelin maintenance, such as lipid synthesis, or fatty acid oxidation, rather than in ATP production [5].

The *m*-AAA protease is a large proteolytic complex in the inner mitochondrial membrane endowed with crucial and pleiotropic roles in mitochondria. It regulates the turnover of respiratory chain subunits [6–8], controls ribosome assembly and thereby mitochondrial translation [9, 10], and affects mitochondrial dynamics [11]. In humans, the *m*-AAA protease is composed of two subunits, paraplegin and AFG3L2, which form either homo-oligomeric (AFG3L2 alone) or hetero-oligomeric (AFG3L2 and paraplegin) hexameric functional complexes [12]. The mouse genome contains a third gene, encoding a functional *m*-AAA protease subunit, *Afg3l1*, which can form either homo-oligomers or hetero-oligomers with AFG3L2 or paraplegin [12].

The discovery that both paraplegin and AFG3L2 are implicated in human neurodegenerative diseases has sparked increasing interest in the *m*-AAA protease. Recessive mutations in *SPG7*, encoding paraplegin, lead to hereditary spastic paraplegia (HSP) [13], a neurodegenerative disease affecting the long corticospinal motor axons, while dominant mutations in *AFG3L2* cause spinocerebellar ataxia type 28 (SCA28) [14], associated with atrophy of the cerebellum. Moreover, a severe phenotype combining features of spastic paraplegia and ataxia associated with myoclonic epilepsy (SPAX5) has been linked to a homozygous mutation in *AFG3L2* [15].

A plethora of dysfunctional pathways have been unravelled in cells when the *m*-AAA protease is depleted, including reduced assembly of respiratory complexes [9, 16, 17], COX deficiency, impaired mitochondrial translation, fragmentation of the mitochondrial network [9], disturbance of mitochondrial anterograde transport [18], and calcium dysregulation [19, 20]. Neurons are extremely susceptible to decreased levels of the *m*-AAA protease, and cannot survive *Afg3l2* deficiency [9]. The role of the *m*-AAA protease in glial cells is so far unknown.

Here, we used an inducible Plp1-CreERT transgenic mouse line to delete *Afg3l2* in a wild-type or *Afg3l1*-null background in adult oligodendrocytes. We found that AFG3L2 deficiency was tolerated by oligodendrocytes for a long time, but ultimately led to late-onset myelin abnormalities and axonal degeneration in the spinal cord. In contrast, deletion of both *Afg3l2* and *Afg3l1*, which completely abolishes the *m*-AAA protease, caused rapid cell death of targeted cells. Our study unravels a crucial role of the *m*-AAA protease in protection against cell death, independent from the metabolic profile of the cell, and demonstrates that different thresholds of *m*-AAA protease activity are required in neurons and glial cells.

## Results

### Deletion of *Afg3l2* in adult oligodendrocytes triggers early-onset mitochondrial morphological abnormalities and late-onset myelin abnormalities

**Fig 1:**
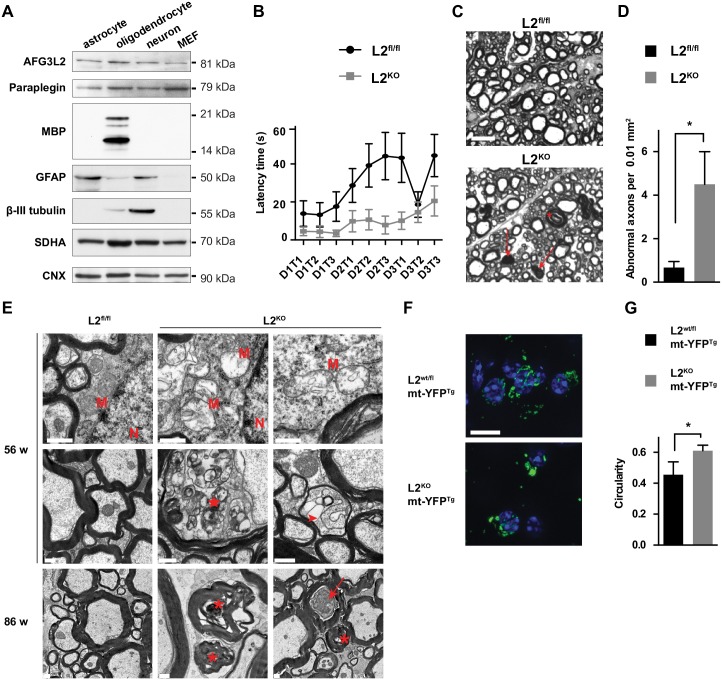
Deletion of *Afg3l2* in oligodendrocytes causes early-onset mitochondrial morphological alterations but late-onset myelin abnormalities. (A) Total lysates (20 μg) from purified astrocyte culture, acutely isolated O4^+^ oligodendrocytes, mixed neuronal-astroglial cultures, and mouse embryonic fibroblasts (MEFs) were probed with the indicated antibodies. GFAP, myelin basic protein (MBP), and β-III tubulin were used as markers of astrocytes, oligodendrocytes, and neurons, respectively. (B) Latency time to fall from a rotarod apparatus of 90-week-old mice (n = 10 mice per genotype). 3 trials (T) per 3 consecutive days (D) were performed. Error bars are SEM. Two-way ANOVA test, p < 0.0001. (C) Representative semithin micrographs of the antero-lateral funiculi spinal cord white matter in 90-week-old mice. Arrows indicate dark degenerating axons and asterisks show thickened myelin. Scale bar, 10 μm. (D) Quantification of abnormal axons (dark degeneration or dysmyelination) per area in semithin sections of 90-week-old mice (n = 3 mice per group). Student’s t-test, p < 0.05. Error bars are SD. (E) Ultrastructural analysis of the lumbar spinal cord white matter in 56- and 86-week-old mice showing adaxonal vacuolization (star), dark axon (arrow), axonal degeneration and myelin disruption (asterisks), myelin detachment (arrowhead), and mitochondrial morphological abnormalities in L2^KO^ mice. M: mitochondria, N: nucleus. Scale bar, 0.5 μm. n = 3 mice per group were analyzed. (F) Representative merged Z-stack images of mitochondria in targeted oligodendrocytes in the corpus callosum visualized by means of a reporter mt-YFP transgene activated by Cre recombination in 8-week-old mice. Immunostaining with anti-GFP antibodies was performed. Nuclei were stained with DAPI. Scale bar, 10 μm. (G) Quantification of mitochondrial shape. A circularity value of 1.0 indicates a perfect circle, while as the value approaches 0, it indicates an increasingly elongated object. We quantified mitochondria in 10–15 cells per mouse in 3 independent mice per genotype. Student’s t-test, p < 0.05. Error bars represent SD.

AFG3L2 is highly expressed in the brain [12], however its abundance in neuronal versus glial cells is unknown. We investigated the expression of subunits of the murine *m*-AAA protease in lysates from enriched neuronal cultures, purified astrocytes, and purified late oligodendrocyte progenitors by immunoblotting. Notably, we did not observe remarkable differences in the levels of AFG3L2 or SPG7 (paraplegin) in neurons versus glial cells (Fig 1A). Minor differences in the expression levels correlated with the abundance of other mitochondrial markers such as SDHA (Fig 1A). Our results are consistent with published quantitative proteomic data obtained from acutely isolated cell population in the brain [21]. These data raise the question of the functional role of the *m*-AAA protease in glial cells.

To target oligodendrocytes, we utilized a well-established tamoxifen-inducible Plp1-CreERT transgenic line [22]. Sensitivity and specificity of the Cre expression was confirmed by crossing Plp1-CreERT^wt/tg^ mice with a reporter transgenic line expressing a mitochondrially targeted YFP (mt-YFP) upon Cre recombination (ROSA26^+/SmY^)[23]. Tamoxifen was injected intraperitoneally for five consecutive days at P29, a time point used in a previous study to induce Cox10 deletion using the same promoter [2], and the corpus callosum analysed at P36 (S1A Fig). mt-YFP expression was largely restricted to cells expressing both or either of the oligodendrocyte markers APC and Olig2 (S1B and S1C Fig). We determined that approximately 50% of oligodendrocytes (positives for either or both APC and Olig2) in the corpus callosum were targeted (S1D Fig).

To induce *Afg3l2* deletion in oligodendrocytes, we crossed Plp1-CreERT mice with mice carrying a floxed allele of *Afg3l2* [9], and used the same protocol described above. We analysed *Afg3l2*^*fl/fl*^ *Plp1-Cre*^*tg/wt*^ mice (referred to as L2^KO^) and compared them with *Afg3l2*^*fl/fl*^ *Plp1-Cre*^*wt/wt*^ mice (L2^fl/fl^), similarly treated with tamoxifen. L2^KO^ mice did not show any apparent phenotype in the cage or weight loss compared to controls up to 90 weeks of age (S2A and S2B Fig). However, at this old age, they displayed a mild but significant impairment in motor performance on an accelerating rotarod test (Fig 1B). We then carefully analysed the brain and the spinal cord of L2^KO^ mice to detect any sign of late-onset pathology. No obvious demyelination was detected up to 90 weeks of age, when we only observed the appearance of few bigger APC^+^ cells in the corpus callosum (S2C Fig). Semithin sections of the spinal cord revealed no alterations at 56 weeks (S2D Fig), but abnormal myelin profiles, characterized by myelin thickening and infoldings, and myelin whorls, indicative of axonal degeneration were visible at 90 weeks (Fig 1C and 1D). Ultrastructural analysis of the spinal cord white matter disclosed a few axons characterized by thin myelin, and others showing adaxonal myelin detachment and vacuolization already at 56 weeks. These abnormalities were more prominent at 86 weeks, when degenerating axons surrounded by damaged myelin or containing accumulation of material were present (Fig 1E). Oligodendrocytes contained enlarged mitochondria with disrupted cristae (Fig 1E and S2E Fig), closely resembling those previously described in *Afg3l2*-deficient neurons [9, 17].

Fragmentation of the mitochondrial network occurs at early time points after deletion of *Afg3l2* in neurons [9], and is caused by activation of the stress protease OMA1, which in turn cleaves the dynamin-like GTPase OPA1, leading to impaired mitochondrial fusion [11]. The functional role of this fragmentation in neurons is unclear, since they die shortly after showing this phenotype. To visualize mitochondrial morphology in targeted oligodendrocytes, we further crossed mice with the mt-YFP (ROSA26^+/SmY^) reporter line [23]. To exclude a toxic effect caused by mt-YFP and/or Cre expression, we used as controls mice haploinsufficient for *Afg3l2*. At 8 weeks of age, oligodendrocytes in the corpus callosum of *Afg3l2*^*fl/+*^ *Plp1-Cre*^*wt/tg*^ ROSA26^+/SmY^ mice had a tubular mitochondrial network, while in absence of *Afg3l2* (genotype: *Afg3l2*^*fl/fl*^ *Plp1-Cre*^*tg/wt*^ ROSA26^+/SmY^) mitochondria appeared swollen and fragmented (Fig 1F and 1G), indicating that the residual *m*-AAA protease is not sufficient to prevent this stress response.

The mt-YFP reporter also allowed us to trace the fate of targeted oligodendrocytes. In the corpus callosum, the total number of targeted mt-YFP^+^ cells and of APC^+^ oligodendrocytes was not significantly changed in absence of AFG3L2 compared to control mice at 56 weeks (S2F Fig), in agreement with the lack of overt demyelination. However, while in control mice almost all mt-YFP^+^ cells were also APC^+^, L2^KO^ mice displayed a significantly increased number of mt-YFP^+^APC^-^ cells (S2G Fig).

Thus, even though lack of *Afg3l2* triggered early-onset pronounced mitochondrial morphology defects, oligodendrocytes survived for long time, and myelin alterations occurred only at very old age.

### *Afg3l1* knock-out mice are viable and do not display overt signs of neurodegeneration

**Fig 2:**
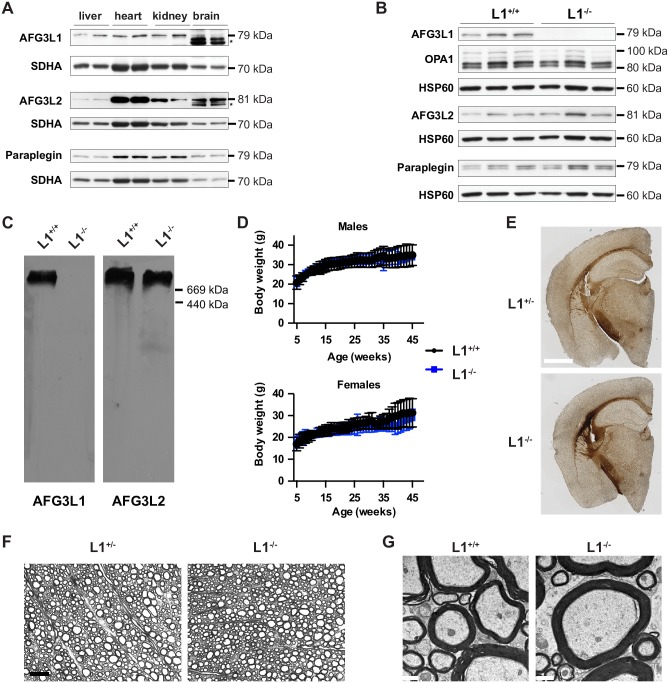
Generation and characterization of *Afg3l1* knock-out mice. (A) Western blot analysis showing AFG3L1, AFG3L2, and paraplegin expression in different tissues. Asterisk indicates an unspecific band. (B) Immunoblot of liver mitochondrial lysates of wild-type (L1^+/+^) and *Afg3l1*^*-/-*^ mice (L1^-/-^) with the indicated antibodies. (C) Blue-native PAGE analysis on mitochondria extracted from the liver and probed with the indicated antibodies revealed lack of assembled *m*-AAA complexes containing AFG3L1 in L1^-/-^ mice. (D) The body weight of L1^-/-^ mice (n ≥ 3) was indistinguishable from their wild type littermates (n ≥ 4) at each age. Error bars are SD. (E) Representative images of Gallyas’ myelin staining of the brain in 50-week-old mice. Scale bar, 1 mm. n = 3. (F) Semithin micrographs of the white matter of the lumbar spinal cord in 50-week-old mice. Scale bar, 20 μm. n = 3. (G) Representative micrographs of the anterolateral funiculus of the lumbar spinal cord in a 62-week-old L1^+/+^ and 78-week-old L1^-/*-*^. Scale bar, 1 μm. n = 3.

The murine *m*-AAA protease subunit AFG3L1 is highly expressed in liver, kidney and heart, but is hardly detectable in the brain (Fig 2A), consistent with previous data [24]. To rule out a major role of *Afg3l1* in the mouse nervous system, we generated a full body knock-out of *Afg3l1*, by deleting exons 2 and 3 (S3A Fig). Analysis of *Afg3l1* transcript levels from liver of 5-week-old mice showed the presence of residual mRNAs after splicing from exon 1 to either exon 4 or exon 5 (S3B Fig). While exon 1–4 splicing gives rise to an out-of-frame transcript, splicing from exon 1 to 5 leads to an in-frame transcript potentially encoding a shorter protein that is devoid of large part of the mitochondrial targeting sequence. However, immunoblotting of liver mitochondria showed no mature AFG3L1 protein (Fig 2B). Most importantly, blue-native PAGE demonstrated lack of assembled AFG3L1 in high-molecular weight *m*-AAA complexes (Fig 2C), confirming that *Afg3l1*^-/-^ mice are *bona-fide* knock-out. AFG3L2 and paraplegin abundance, as well as OPA1 processing, were not affected by lack of AFG3L1 (Fig 2B). *Afg3l1*-deficient mice were born at the expected Mendelian ratio, showed a comparable growth curve to control littermates (Fig 2D), were fertile, and did not show any evident phenotype up to 78 weeks of age. We carefully examined the brain and the spinal cord of *Afg3l1*^*+/-*^ and *Afg3l1*^*-/-*^ mice and detected neither obvious myelination defects nor axonal degeneration at least till 1 year of age (Fig 2F and 2G). Ultrastructural analysis of mitochondria in the spinal cord did not revealed morphological abnormalities (Fig 2G). Thus, in contrast to *Afg3l2*, mouse *Afg3l1* is dispensable in the central nervous system both in neurons and oligodendrocytes.

### Loss of the *m*-AAA protease driven by the Plp1 promoter leads to early-onset motor phenotype and hair greying

**Fig 3:**
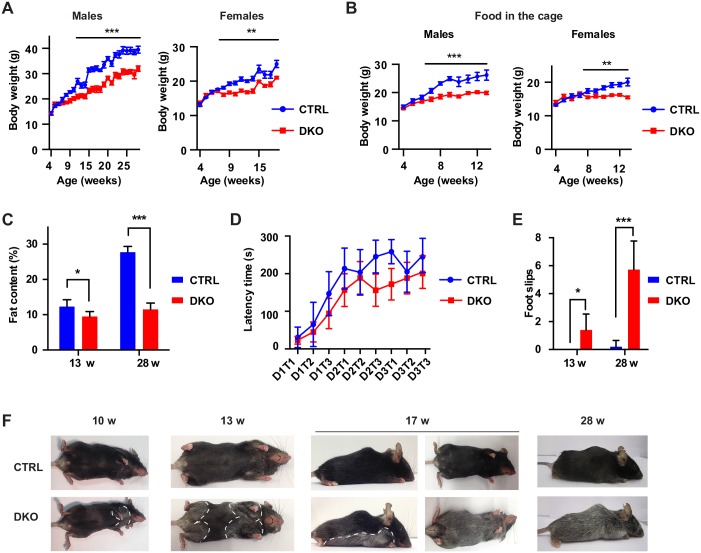
Phenotypic features of DKO mice. (A) Weight curves of DKO mice relative to CTRL mice (males: n ≥ 5 per genotype; females: n ≥ 6 per genotype). Student's t-test, **p < 0.01, ***p < 0.001. Error bars are SEM. (B) Weight curves of DKO mice relative to CTRL mice when food was directly added in the cage (males: n ≥ 6 per genotype; females: n ≥ 8 per genotype). Student's t-test, **p < 0.01, ***p < 0.001. Error bars are SEM. (C) Fat content of mice at 13 weeks (n = 5 per genotype) and at 28 weeks (n = 4 CTRL and 5 DKO) was determined using nuclear magnetic resonance. Student's t-test, *p < 0.05, ***p < 0.001. Error bars are SD. (D) Latency time to fall from a rotarod apparatus of 11–13 weeks old mice. 5 CTRL and 10 DKO mice were tested for 3 trials (T) in 3 consecutive days. Two-way ANOVA test, p < 0.05. Error bars are SEM. (E) Quantification of foot slips during a beam walking test at 13 weeks (n = 5 per genotype) and at 28 weeks (n = 5 CTRL and 7 DKO). Student's t-test, *p < 0.05, ***p < 0.001. Error bars are SD. (F) DKO mice display progressive hair greying.

Paraplegin cannot form homo-oligomeric functional complexes [12], however in absence of AFG3L2 it may assemble together with AFG3L1 and constitute a functional *m*-AAA protease. Therefore, total ablation of *m*-AAA complexes can be achieved in oligodendrocytes by Plp1 promoter-driven recombination of *Afg3l2* in a null *Afg3l1* background. Double knock-out animals (referred to as DKO; genotype *Afg3l1*^-/-^ *Afg3l2*^fl/fl^ *Plp1-Cre*^*tg/wt*^) were compared to control *Afg3l1*^-/-^ littermates (CTRL; genotype *Afg3l1*^-/-^ *Afg3l2*^fl/fl^ *Plp1-Cre*^*wt/wt*^). Cre expression was induced by tamoxifen at 4 weeks, as previously described. Starting from 8 weeks of age, DKO mice failed to gain weight compared to CTRL mice (Fig 3A). This difference persisted even after putting food pellets directly in the cage (Fig 3B). Furthermore, DKO mice had decreased fat mass compared to CTRL mice (Fig 3C). At about 13 weeks, DKO mice started to show signs of motor dysfunction. In a rotarod test DKO mice of 11–13 weeks of age spent less time on the rotating rod compared to CTRL mice (Fig 3D). Moreover, the number of foot slips while walking on a 1 cm-wide beam was significantly increased at 13 weeks, and became dramatically higher at 28 weeks (Fig 3E, S1 and S2 Movies). Surprisingly, DKO mice developed a progressive pattern of hair greying starting ventrally close to the forelimbs at 10 weeks, resulting in a grey belly at 17 weeks, and finally extending to the dorsal skin at 28 weeks of age (Fig 3F).

Thus, concomitant loss of *Afg3l1* in oligodendrocytes strongly exacerbates the phenotypes observed in absence of *Afg3l2*. The DKO mice therefore serve as valuable model to examine the significance of a complete *m*-AAA protease deficiency in myelinating cells.

### Loss of the *m*-AAA protease in adult oligodendrocytes causes progressive demyelination and neuroinflammation

**Fig 4:**
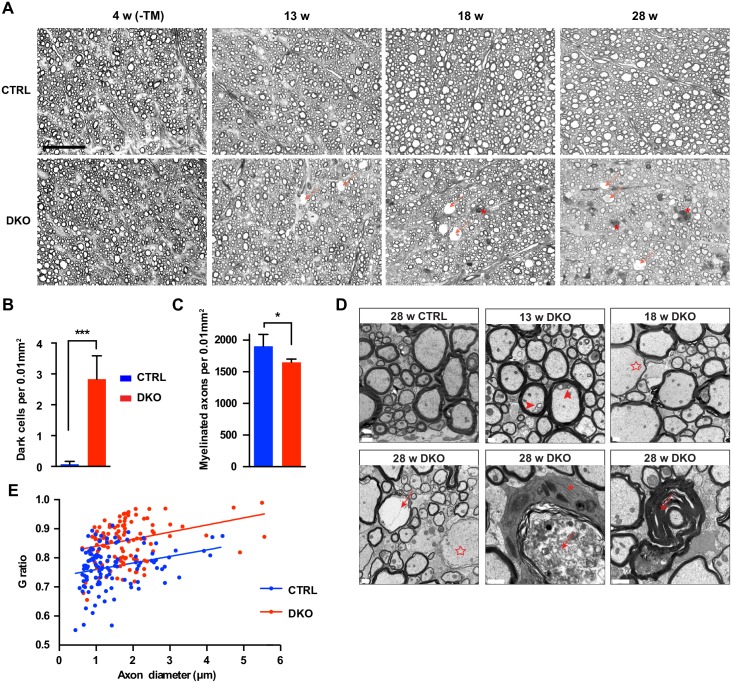
Progressive axonal demyelination in DKO mice. (A) Representative semithin sections of the white matter of the lumbar spinal cord at the indicated time points. Degenerating axons (arrow) and dark cells (asterisk) progressively increase in DKO mice. Scale bar, 50 μm. (B and C) Quantification of dark cells (B) and myelinated axons (C) in the white matter of the spinal cord of 28-week-old mice. Student's t-test, *p < 0.05, ***p < 0.001. Error bars are SD. (D) Electron micrographs of the white matter of the lumbar spinal cord of CTRL and DKO at the indicated ages. Progressive myelin defects and secondary axonal degeneration (arrows) are evident in the spinal cord of DKO mice. Arrowheads indicate adaxonal detachment of myelin, while an arrow with two heads points to myelin folding. Several axons showed thin myelin, a feature of remyelination (empty stars). A dark remyelinating oligodendrocyte surrounding a swollen axon is indicated with an asterisk. Scale bar, 1 μm. n = 3–4 per genotype in A-D. (E) g ratio of axons in the white matter of the lumbar spinal cord of 28-week-old CTRL and DKO mice is plotted according to axon diameter (a total of 118 axons from 3 CTRL and 117 axons from 3 DKO mice were analyzed).

To shed light on the neurological phenotype of DKO mice, we examined brains and spinal cords from DKO and CTRL mice at different time points. At 4 weeks, before tamoxifen injection, the degree of myelination in CTRL and DKO mice was comparable both in the lumbar spinal cord and in the brain (Fig 4A and S4A Fig). However, progressive demyelination was detected in the lumbar spinal cord of DKO, leading to the appearance of demyelinated and degenerating axons as well as dark cells at 28 weeks in the antero-lateral funiculus (Fig 4A–4C). Ultrastructural analysis of the white matter of the spinal cord confirmed progressive demyelination with some axons showing adaxonal detachment of the myelin at 13 weeks, and pronounced signs of demyelination already at 18 weeks (Fig 4D). Signs of secondary axonal degeneration, with accumulation of organelles and material in axons, were visible at 28 weeks (Fig 4D). We identified several axons surrounded by thin myelin, and by oligodendrocytes with dark cytoplasm containing heterogeneous membranous material, probably of lysosomal origin (Fig 4D). These cells have the characteristic of the dark oligodendrocytes, previously proposed to represent mature oligodendrocytes [25, 26]. The g ratio, expressing the ratio between the diameter of the inner axon and the total fiber diameter, was significantly increased at this age (Fig 4E). In agreement with these data, Gallyas’ silver staining of myelinated tracts in the brain showed prominent loss of white matter in the corpus callosum, the internal capsule, and the cerebellum at 28 weeks of age in the DKO mice (S4B Fig).

Progressive loss of myelin was confirmed by western blot analysis of myelin proteins in spinal cord and brain lysates at different time points (S4C and S4D Fig). Concomitantly, we observed upregulation of GFAP, indicating reactive astrogliosis (S4C, S4D and S5 Figs). At 28 weeks, activated microglia cells, which can be recognized by a change in morphology from small cells with slender processes to larger amoeboid-like cells with thick processes, were also detected in the corpus callosum, emphasizing the presence of a neuroinflammatory response (S5 Fig).

### Loss of the *m*-AAA protease triggers death of oligodendrocytes

**Fig 5:**
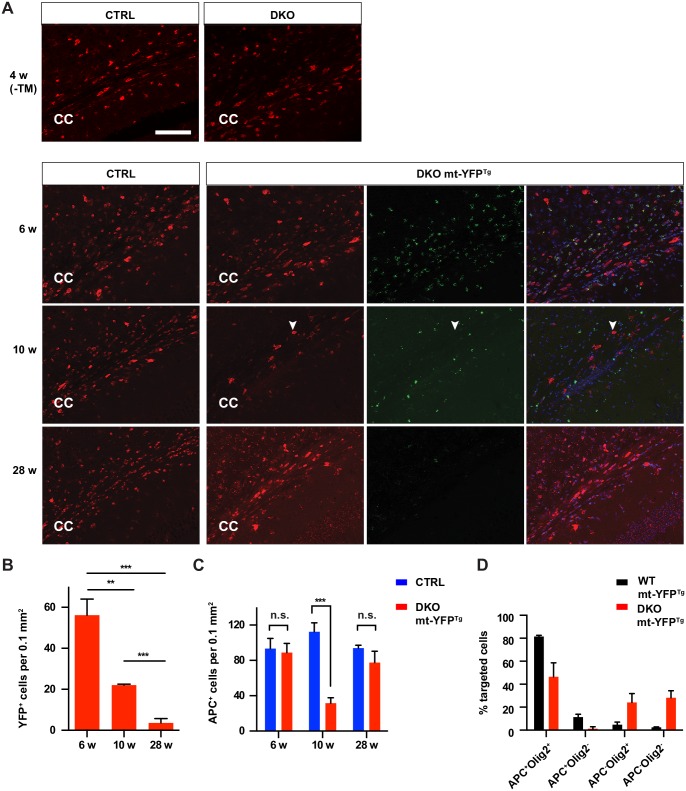
Death of targeted oligodendrocytes and compensatory proliferation of untargeted oligodendrocytes in DKO mice. (A) Representative immunofluorescence merged Z-stack images of total (APC^+^, red) and targeted (mt-YFP^+^, green) oligodendrocytes in the corpus callosum of DKO at the indicated ages. CC: corpus callosum. Arrowhead shows an enlarged APC^+^ mt-YFP^-^ oligodendrocyte. Scale bar, 100 μm. (B) Quantification of mt-YFP^+^ cells in the corpus callosum reveals rapid progressive loss of targeted oligodendrocytes in the DKO. n = 3 mice at each age. Student’s t-test, ** p < 0.01, *** p< 0.001. Error bars are SD. (C) Quantification of total APC^+^ cells in the corpus callosum at different ages. n = 3 mice per group at each time point. t-test, *** p < 0.001. Error bars are SD. (D) Distribution of cells positive for the indicated markers among mt-YFP^+^ cells at 10 weeks of age. Graph shows average of data +/-SD in 3 mice/genotype. chi-square test, p < 0.0001 (743 mt-YFP^+^ cells in WT and 298 in DKO mice were counted).

Since neuroinflammation is a very sensitive read-out of cell damage, we investigated whether cell demise underlies the phenotype. To this end, we crossed DKO mice with the mt-YFP reporter line to visualize targeted oligodendrocytes *in vivo*. Strikingly, mt-YFP^+^ cells were rapidly and progressively lost after 6 weeks, and only a few targeted cells remained at 28 weeks (Fig 5A and 5B). Initially, loss of targeted oligodendrocytes was paralleled by a decrease in APC^+^ cells that was especially evident at 10 weeks both in the corpus callosum and in the spinal cord (Fig 5A and 5C, S6A and S6B Fig). At 10 weeks, the percentage of mt-YFP^+^ cells in the corpus callosum that were also APC^+^ was significantly reduced in DKO mice in comparison with the control line carrying only the mt-YFP reporter, while the percentage of APC^-^ Olig2^-^ targeted cells was significantly increased (Fig 5D). This result is reminiscent of what observed in L2^KO^ mice (S2G Fig).

Surprisingly, the number of mature oligodendrocytes was recovered at 28 weeks in the DKO. This might be explained by the compensatory proliferation and differentiation of untargeted oligodendrocytes that still express *Afg3l2*. Consistently, the size of APC^+^ cells in the DKO mice was increased at 28 weeks (Fig 5A, S6C and S6D Fig), and the enlarged APC^+^ cells did not colocalize with mt-YFP^+^ cells (Fig 5A). We found that the enlarged APC^+^ cells were in fact intensively stained for Olig2 (S6D Fig), a transcription factor expressed at higher levels in migrating and remyelinating oligodendrocytes [27–29]. However, quantification of total Olig2^+^ cells (both intensively and less intensively stained) indicated no statistical difference in the corpus callosum of the CTRL and DKO mice (S6E and S6F Fig).

**Fig 6:**
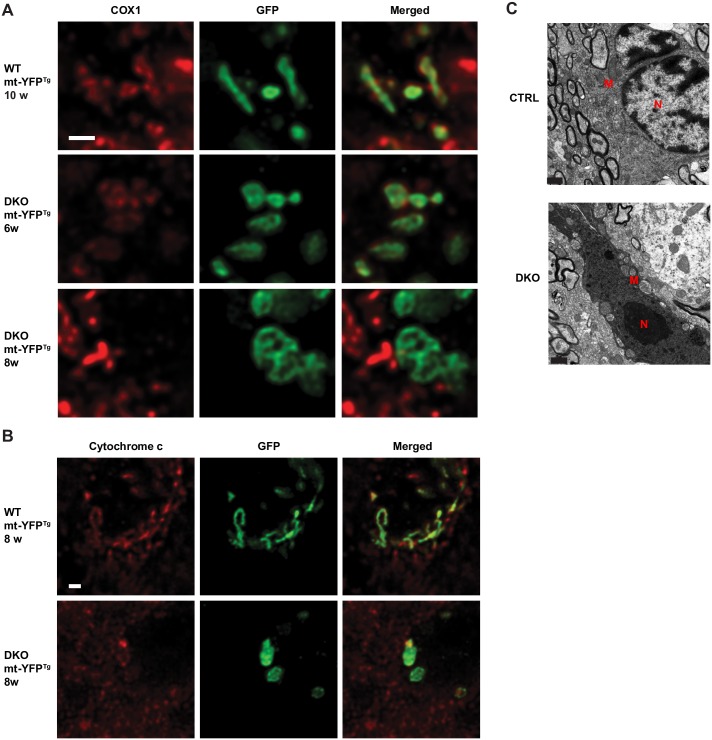
Loss of the *m*-AAA protease in oligodendrocytes causes mitochondrial dysfunction and dark cell death. (A) Deconvoluted single-plane confocal images of double immunofluorescence staining of mt-YFP and COX1 in WT and DKO mice at the indicated age. mt-YFP^+^ mitochondria in DKO lose COX1 staining. Scale bar, 1 μm. (B) Deconvoluted single-plane confocal images of double immunofluorescence staining of mt-YFP and cytochrome c in WT and DKO mice at 8 weeks. Scale bar, 1 μm. (C) Ultrastructural analysis shows an oligodendrocyte undergoing dark cell death in the corpus callosum of 8-week-old DKO mice. M: mitochondria; N: nucleus. Scale bar, 1 μm. n = 3 per genotype in all experiments.

When we monitored mitochondrial morphology, taking advantage of the expression of the mt-YFP reporter in targeted oligodendrocytes, we found abnormal swollen mitochondria already at 6 weeks of age in the DKO mice (Fig 6A). COX1 staining was preserved at this time, but was lost at 8 weeks in targeted oligodendrocytes of the DKO, indicating impairment of mitochondrial respiratory function (Fig 6A). Moreover, cytochrome c was undetectable in several swollen mitochondria in DKO oligodendrocytes at 8 weeks (Fig 6B). We found several oligodendrocytes showing features of dark cell death (Fig 6C), a caspase-independent form of death, characterized by strong cytoplasmic condensation, chromatin clumping, ruffling of the cell membrane, but no blebbing of the nucleus or plasma membrane [30]. Consistently, *in situ* TUNEL assay showed only a few apoptotic cells in DKO mice at 7 weeks of age (quantification in the corpus callosum: 4.13 ± 0.533 in the CTRL mice, 9.75 ± 0.85 in the DKO mice, n = 4 mice per genotype). In summary, these results suggest that the complete loss of *m*-AAA protease causes major mitochondrial dysfunction and death of mature oligodendrocytes followed by compensatory repopulation by untargeted oligodendrocytes.

### Loss of the *m*-AAA protease causes death of melanoblasts and pathology of non-myelinating Schwann cells

One surprising finding was that the DKO mice showed progressive hair greying (Fig 3F). During embryonic development, the Plp1 promoter has been shown to target not only oligodendrocytes, but also Schwann cell (SC) precursors (SCPs), bipotential progenitors of both SCs and melanoblasts [31, 32]. The role of SCPs in the formation of new melanocytes in the adult, and during age-related hair greying remains unknown. Moreover, there is evidence for weak expression of the Plp1 promoter in embryonic melanocytes [33–35], but its activity in adult melanoblasts and melanocytes is unclear. The observed greying of DKO mice thus raised the question whether melanoblasts were targeted and SCPs were affected.

To this end, we performed fate-mapping experiments using the mt-YFP reporter line to establish which cells are targeted in our experiments. We administered tamoxifen at P29 for 5 days, and then collected the ventral or dorsal skin of wild-type mice at P36. This time corresponds to the growth phase called anagen of the second hair cycle in the mouse. During hair follicle (HF) growth, unpigmented melanoblasts differentiate from melanocyte stem cells residing in or close to the hair follicle bulge area and migrate within the outer root sheath of the HF towards the hair matrix where they differentiate into fully mature pigmented melanocytes [36]. We identified mt-YFP^+^ signal in SCs in the subcutaneous nerve plexus or in the nerves surrounding the HFs, in the bulge area containing melanocyte stem cells, in melanoblasts located in the outer root sheath of the HFs, in pigmented bulbar melanocytes (S7 Fig). Remarkably, targeting was more efficient in the ventral than in the dorsal skin, thus providing a potential explanation for the ventral to dorsal progression of hair greying (S7 Fig).

**Fig 7:**
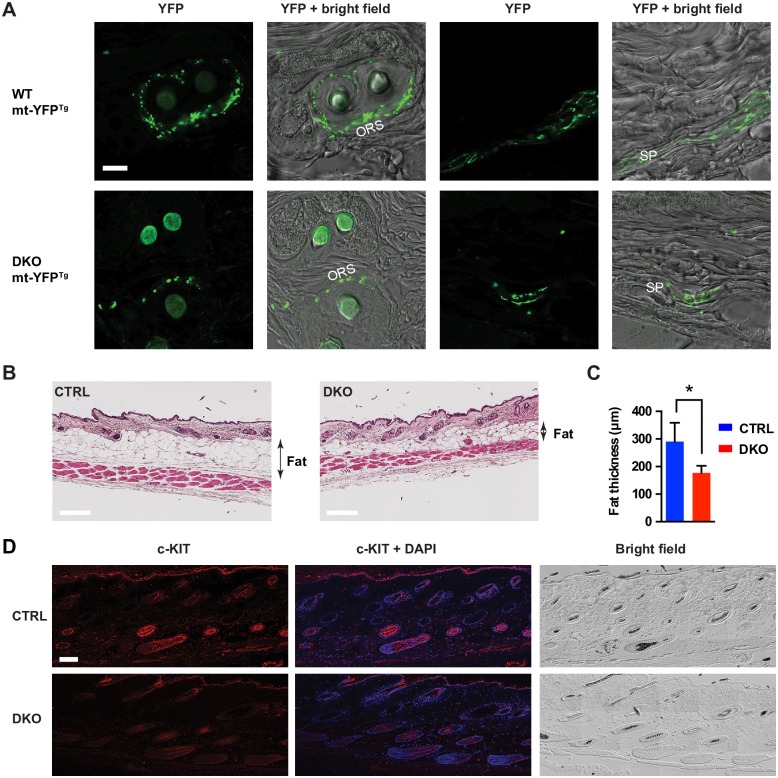
Loss of melanoblasts and melanocytes in DKO mice. (A) Confocal fluorescent and double fluorescent/bright field images of the dorsal skin of 10-week-old WT and DKO mice carrying the mt-YFP transgene show loss of mt-YFP signal in the subcutaneous plexus (SP) and the outer root sheath (ORS). Scale bar, 10 μm. (B) Haematoxylin-eosin staining of dorsal skin of CTRL and DKO mice at 28 weeks. Scale bar, 200 μm (C) Quantification of fat thickness in the dorsal skin. Student’s t-test, p < 0.05. Error bars are SD. n = 3–4 mice/group. (D) Confocal fluorescent and double fluorescent/bright field images of the dorsal skin of 28-week-old CTRL and DKO mice. Several images are assembled using the tile scan function and show a total area of 0.74 mm^2^. c-KIT positive cells and pigmented HFs are largely decreased in DKO mice. Scale bar, 100 μm. At least 3 mice per genotype were used for all experiments.

We then investigated the fate of targeted cells in the skin of DKO mice. At 10 weeks of age, we observed a strong reduction of mt-YFP^+^ melanoblasts in the outer root sheath in DKO mice (Fig 7A). Moreover, non-myelinating SCs in the subcutaneous nerve plexus also showed a significant decrease of mt-YFP signal (Fig 7A). At 28 weeks, although general skin structure was preserved in DKO mice, there was reduced fat deposited in the dermis (Fig 7B and 7C), consistent with the observed general reduction of fat mass in these mice (Fig 3C). Since HF cycling is largely non-synchronized in the mouse at 28 weeks, we shaved the back of the mice, selected pigmented areas of the skin (containing HFs in anagen) for biopsy, and stained sections with antibodies against c-KIT, a marker of melanocytes and melanoblasts. Strikingly, DKO mice showed a significant reduction of pigmented HFs (79.7% ± 2.9 in CTRL versus 28.0% ± 12.3 in DKO mice, n = 3, Student’s t-test, p < 0.05) and c-KIT-positive melanoblasts and melanocytes (Fig 7D). We conclude that hair greying is caused by progressive loss of melanocyte stem cells and melanoblasts that are targeted in our experiments.

**Fig 8:**
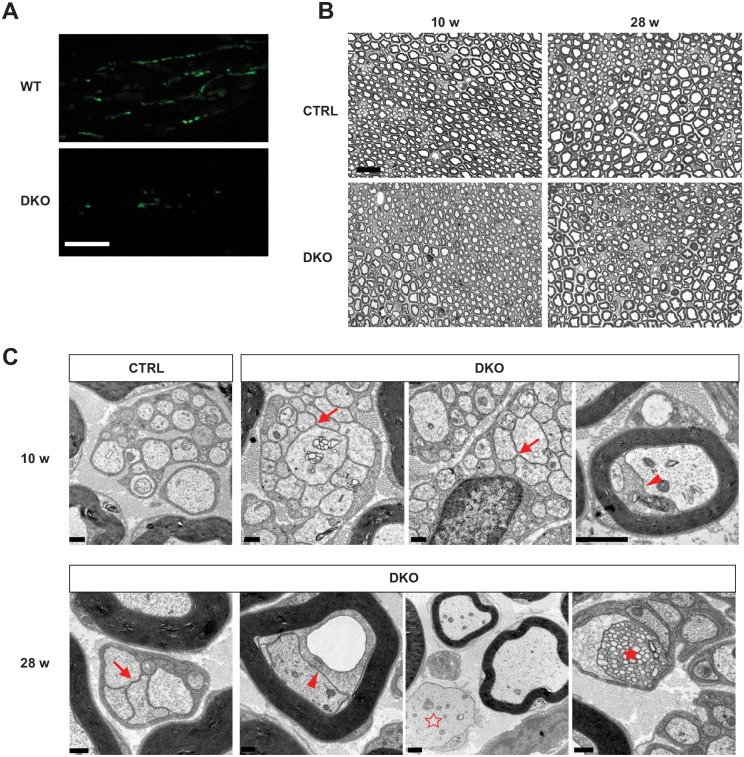
Peripheral neuropathy in the DKO mice. (A) Representative single-plane confocal images showing mt-YFP signal in sciatic nerves at 10 weeks. Scale bar, 10 μm. (B) Representative semithin sections of the sciatic nerves. No overt demyelination was observed in DKO mice. Scale bar, 20 μm. (C) Electron micrographs of the sciatic nerve at 10 weeks and at 28 weeks. While CTRL mice display normal Remak bundles, in DKO mice some unmyelinated axons are touching each other (arrows). Moreover, inner tongue swellings were observed in myelinated axons (arrowheads). In 28-week-old DKO mice non-myelinated large caliber axon (empty star) and multivesicular disintegration of adaxonal myelin lamellae (filled star) were also noted. Scale bar, 1 μm.

Given the fact that DKO mice showed a pathological phenotype in unmyelinated cutaneous nerves, we further examined if peripheral nerves were affected. The Plp1 promoter is known to be expressed at low level in adult SCs [22]. Consistently, we observed scattered mt-YFP signal within the sciatic nerve of wild-type mt-YFP^tg/wt^ mice at 10 weeks of age, and noticed a reduction of this signal in DKO mice (Fig 8A). Semithin sections of the sciatic nerve did not show a remarkable phenotype at 10 and 28 weeks (Fig 8B), probably because of the very low number of targeted cells. We therefore performed ultrastructural analysis and found clear signs of pathology affecting preferentially small calibre unmyelinated fibers (Fig 8C). These fibers are normally associated with non-myelinating SCs in the so-called Remak bundles that contain several axons wrapped by one individual SC. In a normal Remak bundle the cytoplasm of a SC separates individual axons. In the DKO several Remak bundles appeared affected with individual axons touching each other, and showing initial signs of axonal degeneration (Fig 8C). Some alterations were also observed in a few large calibre myelinated axons. In most cases, these were characterized by enlargement of the inner adaxonal tongue, which contained large vacuoles or other material. Similar changes have been previously observed in *Cnp* knock-out mice [37, 38]. These changes were more pronounced at 28 weeks, when also a few demyelinated axons were noted (Fig 8C).

Together, our data establish the vulnerability of both SCs and melanoblasts to loss of the *m*-AAA protease.

## Discussion

Although the *m*-AAA protease in the inner mitochondrial membrane is essential to preserve respiratory activity in neurons, nothing is known about cell autonomous requirements of this complex in glial cells *in vivo*. Here, we have generated mouse models expressing different levels of the *m*-AAA protease in adult myelinating cells. We found that these glial cells survive for long time with reduced levels of the *m*-AAA protease, but total absence of the *m*-AAA protease triggers rapid cell death.

A main conclusion of our study is that the threshold of *m*-AAA protease activity allowing survival of neurons and myelinating cells is remarkably different. This is in line with the fact that mutations in *AFG3L2* or *SPG7* lead to distinct neurodegenerative diseases, characterized by a pure neuronal and axonal phenotype, respectively. What underlies the different cellular vulnerability? We found no significant difference in the expression of AFG3L2 and paraplegin among astrocytes, oligodendrocytes, and neurons, excluding that different stoichiometry of the individual subunits of the *m*-AAA protease plays a crucial role. A possible explanation for our results is the different metabolic profile of adult oligodendrocytes and SCs that are able to survive using glycolysis alone when mitochondrial respiration is impaired. Consistently, oligodendrocyte-specific deletion of an essential assembly factor for complex IV, Cox10, using the same promoter and Cre induction paradigm as in this study, did not lead to axonal degeneration, demyelination, or cell death up to 14 months of age [2]. Moreover, deletion of *Tfam* in SCs caused conspicuous respiratory deficiency, but did not affect their survival [39].

At odds with the hypothesis that oligodendrocytes compensate metabolically for respiratory deficiencies, is the fact that the complete ablation of the *m*-AAA protease is incompatible with cell survival. All targeted cells in the DKO mice, oligodendrocytes, SCs, and melanoblasts, showed mitochondria with dramatically abnormal morphology, and died shortly after removal of the complex. Non-myelinating SCs were affected earlier and more prominently than myelinating SCs, in agreement with previous findings of a peculiar susceptibility of these cells to mitochondrial dysfunction [39]. Oligodendrocytes showed features of dark cell death, very similar to those observed in neurons haploinsufficient for *Afg3l2* [40]. It is conceivable that the respiratory function of mitochondria in post-myelinating oligodendrocytes and SCs is more important than previously thought. Moreover, deficiencies of the *m*-AAA protease likely have more severe effects on the oxidative capacity of the organelles than deletion of *Cox10* or *Tfam*. Indeed, *Cox10* deletion results in isolated complex IV defect, and that depletion of mtDNA upon loss of *Tfam* occurs after a rather long time [41]. In contrast, it was sufficient to ablate *Afg3l2* alone in oligodendrocytes to trigger mitochondrial fragmentation and swelling, a stress response to defective turnover of *de novo* synthesized inner membrane proteins [8, 42], which was not observed in *Cox10*-deficient oligodendrocytes [2]. Consistently, deletion of *Afg3l2* in adult neurons provoked neuronal loss much earlier than observed when a similar strategy was applied to delete *Cox10* or *Tfam* [39, 43], and deletion of *Afg3l2* in oligodendrocytes leads to a late-onset myelin phenotype. In the future, it will be important to develop more genetic models lacking specific mitochondrial proteins involved in respiratory function to fully understand the relevance of oxidative phosphorylation for energy metabolism of adult oligodendrocytes.

An alternative, not mutually exclusive, explanation for the rapid cell demise induced by the lack of the *m*-AAA protease is the activation of a death pathway independent from energy deprivation. We recently found that loss of the *m*-AAA protease results in accumulation of constitutively active MCU-EMRE channels leading to mitochondrial Ca^2+^ overload, mitochondrial permeability transition pore opening and cell death [44]. Such a death pathway would be consistent with the swollen mitochondrial morphology, and the ultrastructural dark appearance of dying neurons and oligodendrocytes depleted of the *m*-AAA protease. Furthermore, the intrinsic vulnerability of neurons to Ca^2+^-dependent cell death may provide a rationale for their increased susceptibility to deficiency of the *m*-AAA protease compared to oligodendrocytes [45].

The fragmentation of the mitochondrial network triggered by *Afg3l2* deletion in oligodendrocytes, as previously observed in neurons [9], raises the question whether this stress-mediated response has different outcomes in oxidative versus glycolytic cells. Emerging data suggest a possible relationship between mitochondrial network morphology and the metabolic capacity of cells [46, 47]. However, in oligodendrocytes, stress-induced mitochondrial fragmentation may be beneficial when transient, but become detrimental if persistent [42], thus contributing to the late-onset myelin abnormalities in mice carrying *Afg3l2* deletion in oligodendrocytes.

In the central nervous system, our DKO model recapitulates features already observed in models of demyelination [48–50]. Loss of the myelin sheaths in DKO mice occurred a few weeks after oligodendrocyte cell death, consistent with previously described long-term stability of the myelin proteins and lipids [51–53], and was associated with a regenerative response of oligodendrocytes leading to some degree of myelin repair, further highlighting the reparative potential of adult oligodendrocytes. However, we cannot completely exclude that part of the phenotype is caused by impaired myelin formation, since myelination is not totally complete at P28 when we injected tamoxifen. A consistent finding, both in L2^KO^ and DKO mice, was an increased percentage of targeted cells that were negative for both APC and Olig2. These cells may represent either oligodendrocytes that lose expression of these markers before dying, and/or oligodendrocyte precursors which fail to differentiate. In fact, experiments in wild-type mice using the mt-YFP reporter line showed that a small percentage of APC^-^ Olig2^-^ cells were targeted shortly after tamoxifen injection, but were hardly detectable at 10 weeks (compare S1C Fig and Fig 5D). Tailored experiments need to be performed in the future to address whether the *m*-AAA protease or a tubular mitochondrial network are required during oligodendrocyte differentiation.

Surprisingly, DKO mice lost weight and fat mass. Albeit we do not know at present the reason for this phenotype, we excluded that this is the result of neurological impairment, hampering to access food. It is conceivable that hypothalamic brain areas involved in feeding behavior may be affected by demyelination. Furthermore, we cannot rule out the possibility that the Plp1 promoter is expressed in other cell types than myelinating cells, contributing to this phenotype.

An unexpected phenotype in DKO mice was premature and progressive hair greying. Melanocytes and SCs both arise from the neural crest. A previous study has identified both in chick and mouse two distinct migratory pathways producing melanocyte stem cells during development. At E10-E11.5, melanoblasts delaminate from the murine neural tube and migrate dorsally between the dermomyotome and the epidermis to populate the skin. Later, at E12-E14 bipotential precursors of both melanoblasts and SC, the SCPs, leave the neural crest along a ventral migratory route along the nerves [31, 32]. According to these studies, at around E13, SCPs detaching from the nerve differentiate into melanoblasts and downregulate Plp1, while those that remain attached to the nerve acquire SC properties [31]. This second migratory wave would contribute to melanoblasts in the limbs, the belly, and the dorsal skin, and has been identified performing fate-mapping experiments using a Plp1-CreERT2 transgenic line [31]. In our hands, at 4 weeks the Plp1 promoter is active not only in the nerve, but also in cells that are located in the bulge area and in the outer root sheath of the HF, and therefore have lost contact with the nerve, suggesting that they are melanoblasts. Importantly even a few melanocytes were targeted, as others have observed at developmental stages [34]. We therefore conclude that the Plp1-CreERT line is not suitable to determine whether SCPs contribute to melanocytes in the adult mouse. Aging-associated hair greying has been linked to increased differentiation and/or loss of melanocyte stem cells [36]. As in our model stem cells are lost, without an initial increase in differentiated pigmented cells, the latter is the most likely mechanism, coupled with the concomitant death of targeted melanocytes. Premature hair greying has been previously reported in the mutator mouse, a genetic model of accelerated aging caused by expression of a proof-reading-defective mitochondrial Polg DNA polymerase [54]. The mechanism of hair greying in this model has not been investigated in detail, and more studies are needed to understand the role played by mitochondrial dysfunction or intrinsic pathway of apoptosis in aging-related hair greying.

Finally, we show here that constitutive deletion of *Afg3l1* alone in the mouse does not lead to an evident neurological phenotype. This is consistent with the extremely low levels of expression in the brain. However, our study indicates that AFG3L1 can largely rescue deficiency of AFG3L2 in oligodendrocytes, by sustaining residual *m*-AAA activity likely in complex with the more abundant paraplegin subunit. Since *Afg3l1* is a functional gene in the mouse but not in humans [55], examining the phenotypic consequences of mutations in *Afg3l2* or *Spg7* in an *Afg3l1* null background now offers a more suitable model mimicking the human situation.

In summary, our data shed new light on functional requirements of the mitochondrial *m*-AAA protease in adult oligodendrocytes, help understanding cell-specificities in the context of the human pathologies, and provide insights in oligodendrocyte, SC, and melanoblast survival mechanisms.

## Materials and Methods

### Mouse experiments

All animal procedures were carried out in accordance with European (EU directive 86/609/EEC), national (TierSchG), and institutional guidelines and were approved by local authorities (Landesamt für Natur, Umwelt, und Verbraucherschutz Nordrhein-Westfalen, Germany; approval numbers 87–51.04.2010.A219 and 84–02.04.2015.A402). Plp1-CreERT mice [22] were purchased from Jackson Laboratory. Conditional *Afg3l2*^fl/fl^ mice [9] and ROSA26^+/SmY^ mice [23] were previously reported. *Afg3l1*^-/-^ mice were commercially generated in C57BL/6N background at Taconic-Artemis. Plp1-CreERT mice were mated with ROSA26^+/SmY^ mice to visualize mitochondria in oligodendrocytes. To obtain L2^KO^ mice, *Afg3l2*^fl/fl^ mice were crossed to Plp1-CreERT mice. As controls, we used *Afg3l2*^fl/fl^ Cre-negative littermates. Mice of both genotypes were injected with tamoxifen as specified below. *Afg3l1*^-/-^*Afg3l2*^fl/fl^ *Plp1*-Cre^wt/tg^ (DKO) mice were compared with *Afg3l1*^-/-^*Afg3l2*^fl/fl^*Plp1*-Cre^wt/wt^ (CTRL) littermates injected with tamoxifen. To investigate mitochondrial morphology in L2^KO^ mice or in DKO mice, when indicated, *Afg3l2*^fl/fl^*Plp1*-Cre^wt/tg^ or *Afg3l1*^-/-^*Afg3l2*^fl/fl^*Plp1*-Cre^wt/tg^ mice were crossed with ROSA26^+/SmY^ mice. Tamoxifen (T5648, Sigma) was dissolved in a corn oil/ethanol (9:1) mixture at a final concentration of 10 mg/ml. 1 mg tamoxifen was administrated by intraperitoneal injection once a day for 5 consecutive days to 4-week-old (P28-30) mice. Unless specified, mice of either sex were used for experiments.

### Behavioral tests

The rotarod apparatus (TSE systems) was used to test motor ability and coordination. Mice were placed on a rotating rod (accelerating model) and the latency time to fall was recorded for each mouse up to a maximum of 300 seconds. Three tests were performed for three consecutive days. Mice were allowed to rest for 15 minutes after each test. In the beam walking test, mice were trained to walk on a 90 cm long and 3 cm wide beam, elevated by 30 cm on a metal support, for three times for three consecutive days. The actual test was performed by allowing the mice to walk on a 1 cm wide beam on the third day. The performance was filmed and the number of foot slips was quantified. The lean and fat mass of mice was measured with Bruker Minispec Live Mice Analyzer (LF50H).

### Tissue collection

Mice were deeply anesthetized with xylazine/ketamine (10 mg/100 mg per kg of body weight) and perfused transcardially with PBS and 4% paraformaldehyde (PFA). Brain, spinal cord, and the peripheral nerves were then dissected and postfixed in 4% PFA for histology and immunofluorescence or in 2% glutaraldehyde in 0.12 M phosphate buffer for electron microscopy. The skin was collected after shaving the mice and immersed in 4% PFA for 2–4 h at 4°C. For RNA extraction, western blot analyses and TUNEL assay, mice were sacrificed by cervical dislocation.

### RNA extraction and RT-PCR

Tissues were quickly collected and frozen in liquid nitrogen. RNA extraction was performed with TRIzol reagent (Life Technologies) according to the manufacturer specifications. cDNA was synthesized using SuperScript First-Strand Synthesis System (Life Technologies). The sequence of primers used for RT-PCR are available upon request.

### Gallyas’ and immunofluorescence stainings

Postfixed brain and spinal cord were embedded in 6% agar, and 30 μm sections were cut using a vibratome (VS1000, Leica). Gallyas’ staining was performed as previously described [56] and images were captured with slide scanner (SCN400, Leica). For immunofluorescence, free-floating sections were permeabilized and blocked in 0.4% Triton X-100 and 10% goat serum in TBS for one hour at RT. Primary antibodies were incubated overnight at 4°C, followed by incubation with secondary antibodies for 2 h at RT. Sections were mounted in FluorSave Reagent (Calbiochem). Skin specimens were embedded in paraffin and sectioned at 5 μm thickness using a microtome (RM2255, Leica). Skin sections were stained with Haematoxylin solution (MHS32, Sigma) and Eosin Y-solution 0.5% aqueous (1098441000, Millipore). Antigen retrieval was conducted by boiling sections in 0.1 M citrate buffer (pH 6) before immunofluorescence analysis. The following primary antibodies were used for immunofluorescence: APC (1:400, OP80, Calbiochem), COX1 (1:1000, 459600, Invitrogen), cytochrome c (1:1000, 556432, BD Pharmingen), Olig2 (1:500, AB9610, Millipore), GFAP (1:400, 3670, Cell Signaling), IBA1 (1:2000, 019–19741, Wako), GFP (1:1000, ab6556, Abcam), MBP (1:1000, SMI94, Covance), and c-KIT (1:1000, 553352, BD Pharmingen). All secondary antibodies were from Molecular Probes: anti-mouse Alexa Fluor 488 (A-11029), 546 (A-21143), anti-rabbit Alexa Fluor 488 (A-11034), 546 (A-11035), 594 (A-21207), and anti-rat Alexa fluor 488 (A-11001). All fluorescent images were acquired using an Axio-Imager M2 microscope equipped with Apotome 2 (Zeiss) or gSTED super-resolution and confocal microscope with HyD detector (TCS SP 8, Leica), as specified. When specified, Huygens Deconvolution software was employed.

### Quantification of cell number in immunofluorescence experiments

Quantification of APC^+^, Olig2^+^, and mt-YFP^+^ cells was performed manually on single plane images of brain coronal vibratome sections (30 μm). Three sections of each brain cut at comparable levels (about -1.50 mm, -2.8 mm, -3.4 mm from the bregma) were stained with the indicated antibodies. Two to five images of non-overlapping fields in the corpus callosum of one hemisphere were taken for each section and the number of positive cells/area was manually counted. 3 independent mice per genotype and time point were used for quantification. Quantification of APC^+^ cell size was performed on images acquired using the same exposure time using the measure function of the Axiovision software (Zeiss).

### Mitochondrial morphology

To visualize mitochondrial morphology in targeted cells, mice were crossed with ROSA26^+/SmY^ mice. To visualize endogenous mt-YFP signal, sciatic nerves were dissected out, embedded in O.C.T. (Tissue-Tek) and were cut longitudinally at a thickness of 7 μm using a cryostat (CM1850, Leica). For cryosections, the skin was cryoprotected in 15% sucrose for 2 h and then in 30% sucrose overnight, embedded in O.C.T. (Tissue-Tek), frozen on dry ice and sectioned with a cryostat (CM1850, Leica). 10 μm frozen sections were directly mounted for imaging. For all analyses performed in the brain, vibratome sections were incubated with an anti-GFP antibody to enhance the endogenous YFP signal. Fluorescent images were acquired using a gSTED super-resolution and confocal microscope with HyD detector (TCS SP 8, Leica), as specified. The circularity of mitochondria in targeted oligodendrocytes within the corpus callosum was measured using a macro of ImageJ. The circularity was calculated with the following formula: 4 *pi*(area/perimeter^2) [57].

### TUNEL assay

To detect TUNEL^+^ cells, the *In Situ* Apoptosis assay (S7101, Millipore) was used on cryostat sections out following the manufacturer’s protocol. The number of TUNEL^+^ cells within the corpus callosum was quantified manually. 4 mice per genotype and 2–3 sections from each mouse were used for quantification.

### Semithin section and electron microscopy

The corpus callosum and the lumbar spinal cord were post-fixed in 2% glutaraldehyde (Sigma) in 0.12 M phosphate buffer and were treated with 1% osmium tetroxide (Sigma). After dehydration with ethanol and propylene oxide, tissues were embedded in Epon (Fluka). Tissue in Epon-block was further trimmed and cut using an ultramicrotome (EM UC7, Leica). 1 μm semithin sections were prepared and stained with 1% toluidine blue for light microscopy. For electron microscopy, 70 nm ultrathin sections were cut and stained with uranyl acetate (Plano GMBH) and lead citrate (Electron Microscopy Sciences). Images were taken by a transmission electron microscope (CM10, Phillips) equipped with Orius SC200W camera.

### Quantification in semithin and ultrathin sections

Quantification of dark cells and number of myelinated axons was performed on at least three semithin micrographs of the anterolateral funiculus of the lumbar spinal cord per mouse. Three independent mice per genotype were analyzed. The number of myelinated axons was quantified using ImageJ particle analyzer with the setting of size 50-infinity and circularity 0.3–1.0. The g ratio was determined by measuring the ratio between the diameter of the axon and the diameter of the myelinated fiber on electron micrographs from 3 mice per genotype.

### Oligodendrocytes preparation

Brains from P5 pups were removed and manually dissociated with the Neuronal Tissue Dissociation Kit (130-092-628, Miltenyi Biotec). To purify oligodendrocytes, the cell suspension was further incubated with anti-O4 magnetic beads (130-096-670, Miltenyi Biotec), washed and loaded onto 30 μm pre-separation filters fixed on top of MS MACS Columns (130-042-201, Miltenyi Biotec), which were placed in a magnetic field of the MACS separator (Miltenyi Biotec). The magnetic labeled O4^+^ cells were retained within the columns, while flow-through was collected. Finally, magnetically labeled cells were flushed out by firmly pushing the plunger into each column.

### Astrocyte and enriched neuronal cultures

Astrocytes were isolated from the cerebellum of P0-P3 newborn pups. The cerebellum was dissected in dissection solution (60% EBSS; 4% Glucose; 30 mM HEPES; 30% FCS III). After removing the meninges, the tissue was mechanically meshed, washed twice with EBSS solution (10% HEPES; 90% EBSS), and placed in glia medium (90% DMEM/F12 Hams media with L-glutamine; 9% FCS III; 1% Pen/Strep). The single-cell suspension was obtained by mechanical dissociation. The cells were then passed through a 100 μm Nylon cell strainer and the strainer was then washed with 5 ml of glia medium. The cells were centrifuged at 800g for 5 minutes at 4°C. The supernatant was removed and the pellet was resuspended in 10 ml glia medium and plated in 75 cm^2^ flasks previously coated with poly-L-lysine (0.1mg/ml). For enriched neuronal cultures, the cortex and the hippocampus were dissected from E16.5 mouse embryos. The meninges were removed prior mechanical dissociation of the tissue. Chemical dissociation was obtained with Trypsin solution (0.025% in HBSS) for 15 minutes at 37°C. The tissue was then washed 3 times with HBSS for 5 minute at 37°C and triturated with Pasteur pipettes (0.5 mm opening size) for 7 times. The cells were cultured in in Neurobasal plating Media. One week following the seeding, the cells were collected for protein extraction.

### Mitochondria isolation and blue native polyacrylamide gel electrophoresis

Mitochondria from tissues were isolated in MOPS sucrose buffer (440 mM sucrose, 20 mM MOPS, 1 mM EDTA, 0.2 mM phenylmethylsulfonyl fluoride) by differential centrifugation at 10,000 g. 100 μg of mitochondria were solubilized in 1 M ε-amino n-caproic acid, 50 mM Tris (pH 7.0) and digitonin at a detergent to protein ratio of 4 g/g. BN-PAGE was performed as previous described [58].

### Western blot analysis

Cells or tissues were lysed in RIPA buffer and immunoblot analysis was conducted as described [18]. The following primary antibodies were used: AFG3L1 (1:1000) [12], AFG3L2 (1:1000) [12], Paraplegin (1:500) [59], SDHA (1:4000, A11142, Molecular Probes), β-III tubulin (1:1000, T8660, Sigma), MBP (1:2000, SMI94, Covance), CNP (1:500, C5922, Sigma), GAPDH (1:2000, MAB374, Chemicon), CNX (1:4000, ADI-SPA-860, Enzo), GFAP (1:2000, Z0334, Dako).

### Statistical analysis

All statistical analyses were performed using GraphPad Prism 6 software, presenting the data as mean ± standard deviation (SD) or as mean ± standard error of the mean (SEM). If not stated otherwise, *p* value was determined by two-tailed unpaired Student’s *t* test. Statistical significance was defined as *p < 0.05, **p < 0.01 and ***p < 0.001.

## Supporting Information

S1 Fig Tamoxifen-induced recombination in adult *Ppl1-CreERT*^*+/tg*^ *ROSA26*^*+/SmY*^ mice. (A) Schematic representation of the timeline of tamoxifen injection and analysis. (B) Coronal sections across the corpus callosum from *Plp1-CreERT2*^*+/tg*^ *ROSA26*^*+/SmY*^ mice analyzed at P36 were stained with APC, Olig2, and GFP antibodies. Scale bar, 20 μm. (C) Quantitative analysis of the specificity of recombination in the corpus callosum. Distribution of cells positive for the indicated markers among mt-YFP^+^ cells. (n = 3) Error bars are SD. (D) Quantitative analysis of the efficiency of recombination in oligodendrocytes (identified as APC^+^ and/or Olig2^+^) the corpus callous. (n = 3). Error bars are SD.
(TIF)

S2 Fig Characterization of L2^KO^ mice. (A) The body weight of L2^fl/fl^ and L2^KO^ mice at 56 weeks of age (females: L2^fl/fl^ n = 7, L2^KO^ n = 6; males: L2^fl/fl^ n = 4 mice, L2^KO^ n = 3 mice), and (B) at 90 weeks of age (females: L2^fl/fl^ n = 6, L2^KO^ = 3; males: L2^fl/fl^ n = 3 mice, L2^KO^ n = 4 mice). (C) Immunostaining of brain coronal sections across the corpus callosum of mice at 90 weeks of age. Oligodendrocytes are stained with APC and counterstained by DAPI. Myelin is stained with a MBP antibody. (D) Representative semithin micrographs of the white matter in the lumbar spinal cord show comparable myelination and axonal integrity in 56-week-old L2^fl/fl^ and L2^KO^ mice (n = 3 per genotype). Scale bar, 20 μm. (E) Low magnification images corresponding to upper row left and middle images of Fig 1E, respectively. Scale bar, 0.5 μm. (F) Quantification of the number of YFP^+^ and APC^+^ cells in the corpus callosum of 56-week-old mice. n = 3 mice per genotype. (G) Quantification of the number of YFP^+^ APC^-^ cells in the corpus callosum of 56-week-old mice. n = 3 mice per genotype. Student’s t-test, p < 0.01. In all graphs error bars are SD.
(TIF)

S3 Fig Generation of *Afg3l1*^-/-^ mice. (A) Gene targeting strategy. (B) RT-PCR on liver cDNA using primers specific for *Afg3l1* located in different exons. No amplification was detected in *Afg3l1*^*-/-*^ mice with primers located either on exon 2 or exon 3. Two bands were amplified when using primers spanning the deletion, representing splicing from exon 1 to either exon 4 or exon 5.
(TIF)

S4 Fig Progressive demyelination and inflammation in DKO mice. (A) Gallyas’ myelin staining of the forebrain at 4 weeks, before tamoxifen injection. Scale bar, 1 mm. (B) Gallyas’ myelin staining of the forebrain and cerebellum in 28-week-old mice. Arrowheads point to myelinated tracts. Scale bar, 1 mm. (C, D) Representative western blots and quantification of brain and spinal cord lysates of DKO mice at 10 weeks (10 w; n = 3 per genotype) and 28 weeks (28 w; n = 4–6 per genotype) of age. Student’s t-test, *p < 0.05, **p < 0.01, ***p < 0.001. Error bars are SD.
(TIF)

S5 Fig Depletion of the *m*-AAA protease in oligodendrocytes is associated with neuroinflammation. Immunofluorescence staining of MBP, GFAP, and IBA1 in the corpus callosum (CC) of CTRL and DKO mice at the indicated ages. Demyelination in the DKO mice is associated with astrocyte and microglia activation. Scale bar, 100 μm.
(TIF)

S6 Fig Quantification of oligodendrocytes in the CNS. (A, B) Immunofluorescence staining and quantification of APC^+^ cells in the dorsal column of the spinal cord of 10-week-old CTRL and DKO mice. n = 3 mice per genotype. Student’s t-test, p < 0.01. Error bars are SD. (C) Quantification of the size of APC^+^ oligodendrocytes in the corpus callosum in 28-week-old mice. n = 3 mice per genotype (244 oligodendrocytes in the CTRL and 205 oligodendrocytes in the DKO were measured). Student’s t-test, p < 0.05. Error bars are SD. (D) Double immunofluorescence staining of APC and Olig2 in the corpus callosum of CTRL and DKO mice at 28 weeks. The enlarged APC^+^ oligodendrocytes in the DKO are more intensively stained by Olig2. Scale bar, 50 μm. (E) Immunofluorescence staining of Olig2^+^ cells in the corpus callosum (CC) of CTRL and DKO mice at the indicated age. n = 3 mice per genotype. (F) Quantification of Olig2^+^ cells in the corpus callosum (CC) of CTRL and DKO mice at the indicated age. n = 3 mice per genotype. Error bars are SD.
(TIF)

S7 Fig Fate mapping experiments in the skin using *Plp1-CreERT*^*+/tg*^ *ROSA26*^*+/SmY*^ mice. (A, B) Single-plane confocal images of dorsal (A) and ventral (close to forelimbs) (B) skin sections from Plp1-CreERT^+/tg^ ROSA26^+/SmY^ mice. Mice were injected with tamoxifen at P29 for five consecutive days and the skin was collected at P36. Endogenous mt-YFP^+^ signal in the cryosections is shown in green. SP: subcutaneous plexus; SG: sebaceous glands; BG: bulge area; DCP: deep cutaneous plexus; MC: melanocytes; ORS: outer root sheath. Scale bar, 10 μm.
(TIF)

S1 Movie Performance of a 28-week-old CTRL mouse on a walking beam. (MP4)

S2 Movie Performance of a 28-week-old DKO mouse on a walking beam. (MP4)
