# Supplementary figures and images for: The Mitochondrial m-AAA Protease Prevents Demyelination and Hair Greying
Source: PLoS Genet. 2016 Dec 2;12(12):e1006463. doi: 10.1371/journal.pgen.1006463 (PMC5135034; doi:10.1371/journal.pgen.1006463)

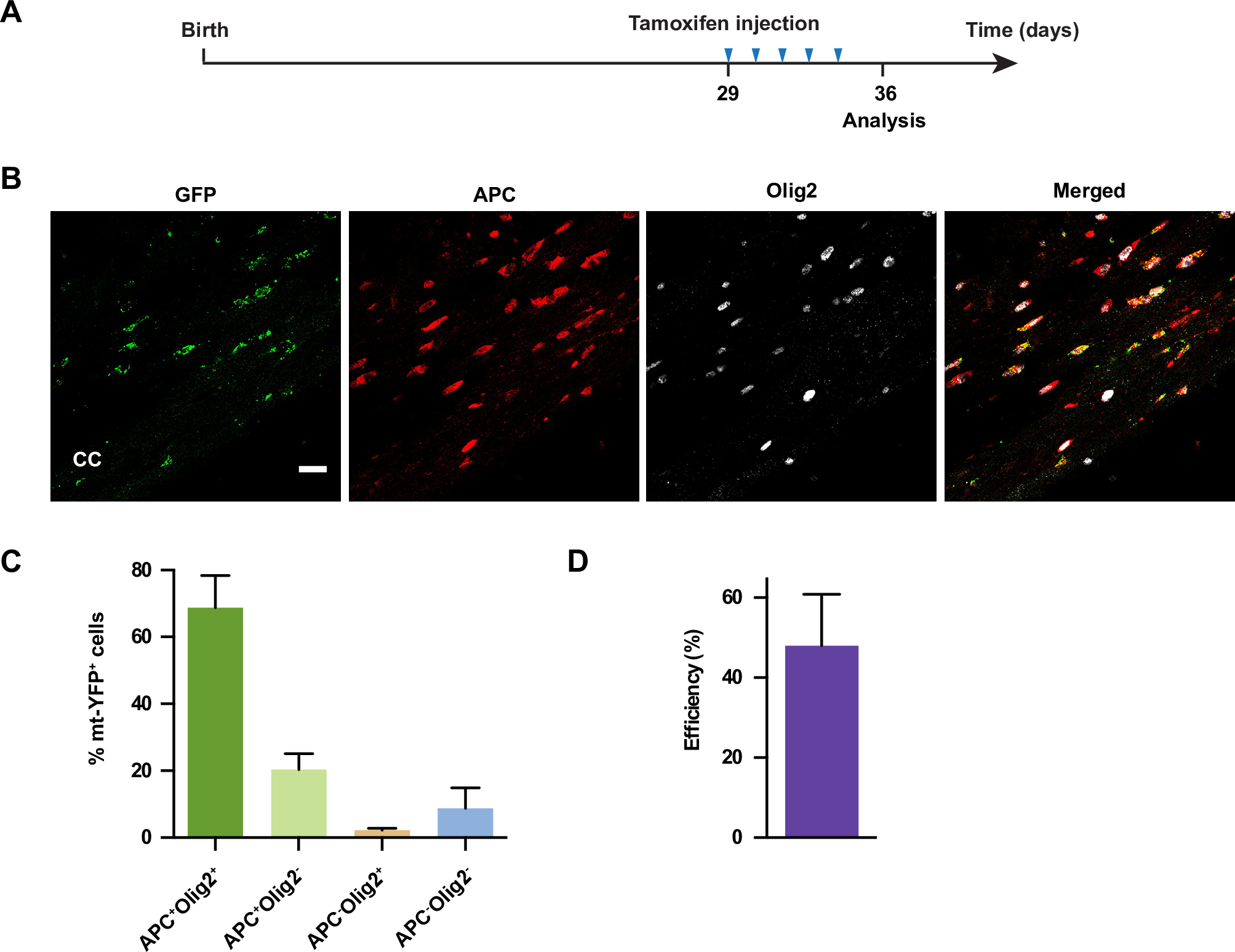

Supplement: S1 Fig — (A) Schematic representation of the timeline of tamoxifen injection and analysis. (B) Coronal sections across the corpus callosum from Plp1-CreERT2+/tg ROSA26+/SmY mice analyzed at P36 were stained with APC, Olig2, and GFP antibodies. Scale bar, 20 μm. (C) Quantitative analysis of the specificity of recombination in the corpus callosum. Distribution of cells positive for the indicated markers among mt-YFP+ cells. (n = 3) Error bars are SD. (D) Quantitative analysis of the efficiency of recombination in oligodendrocytes (identified as APC+ and/or Olig2+) the corpus callous. (n = 3). Error bars are SD. (TIF) [file pgen.1006463.s001.tif]

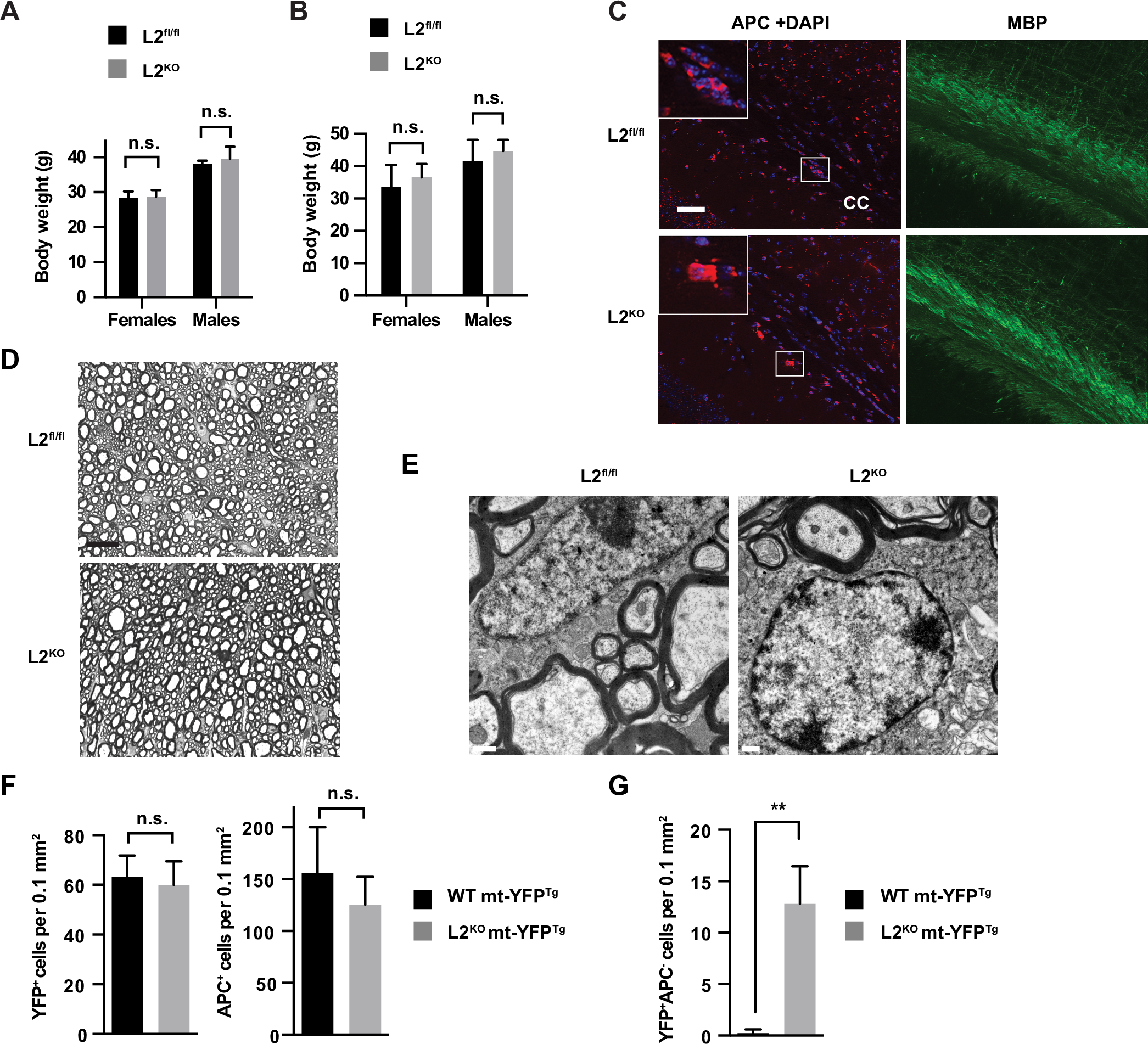

Supplement: S2 Fig — (A) The body weight of L2fl/fl and L2KO mice at 56 weeks of age (females: L2fl/fl n = 7, L2KO n = 6; males: L2fl/fl n = 4 mice, L2KO n = 3 mice), and (B) at 90 weeks of age (females: L2fl/fl n = 6, L2KO = 3; males: L2fl/fl n = 3 mice, L2KO n = 4 mice). (C) Immunostaining of brain coronal sections across the corpus callosum of mice at 90 weeks of age. Oligodendrocytes are stained with APC and counterstained by DAPI. Myelin is stained with a MBP antibody. (D) Representative semithin micrographs of the white matter in the lumbar spinal cord show comparable myelination and axonal integrity in 56-week-old L2fl/fl and L2KO mice (n = 3 per genotype). Scale bar, 20 μm. (E) Low magnification images corresponding to upper row left and middle images of Fig 1E, respectively. Scale bar, 0.5 μm. (F) Quantification of the number of YFP+ and APC+ cells in the corpus callosum of 56-week-old mice. n = 3 mice per genotype. (G) Quantification of the number of YFP+ APC- cells in the corpus callosum of 56-week-old mice. n = 3 mice per genotype. Student’s t-test, p < 0.01. In all graphs error bars are SD. (TIF) [file pgen.1006463.s002.tif]

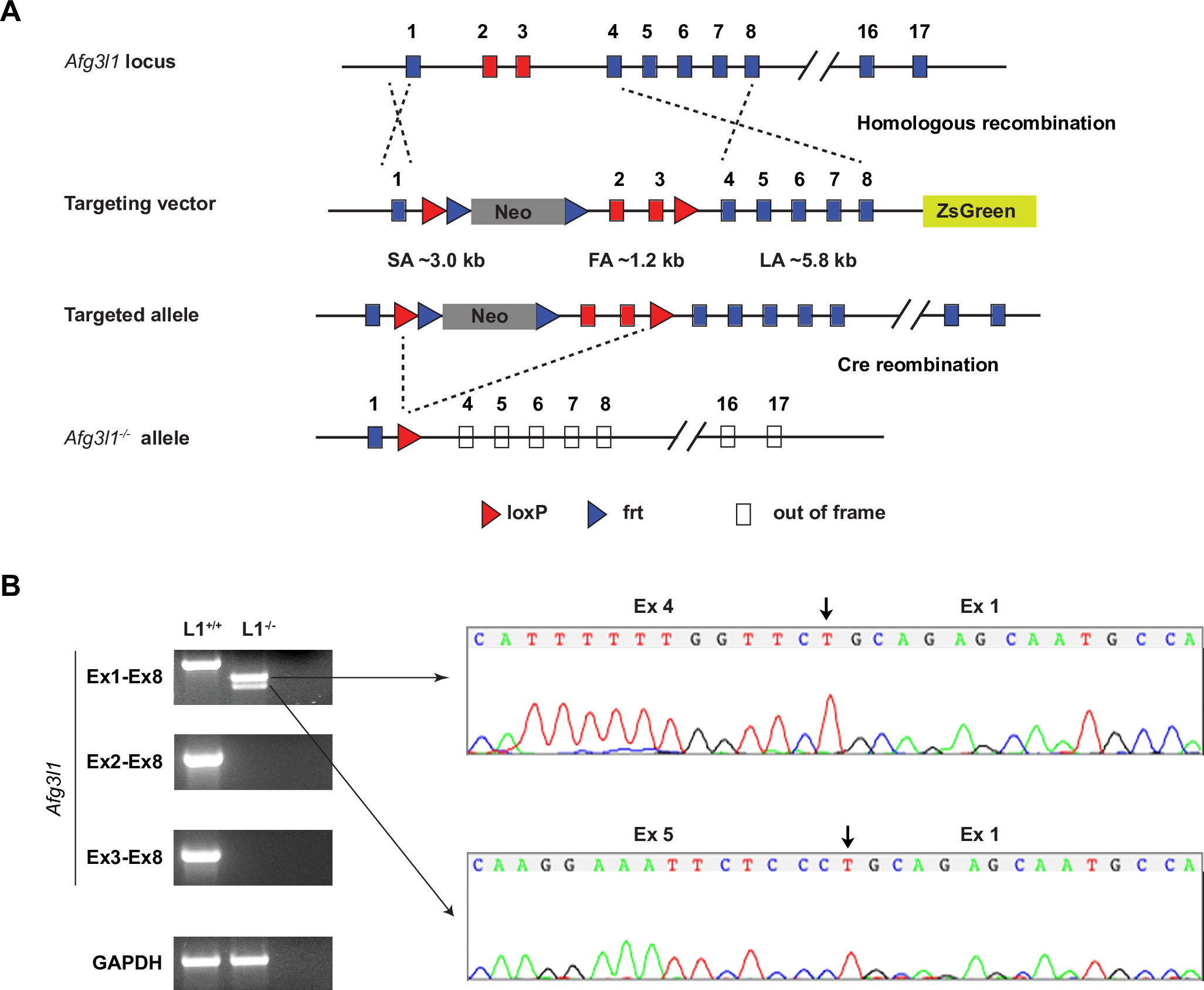

Supplement: S3 Fig — (A) Gene targeting strategy. (B) RT-PCR on liver cDNA using primers specific for Afg3l1 located in different exons. No amplification was detected in Afg3l1-/- mice with primers located either on exon 2 or exon 3. Two bands were amplified when using primers spanning the deletion, representing splicing from exon 1 to either exon 4 or exon 5. (TIF) [file pgen.1006463.s003.tif]

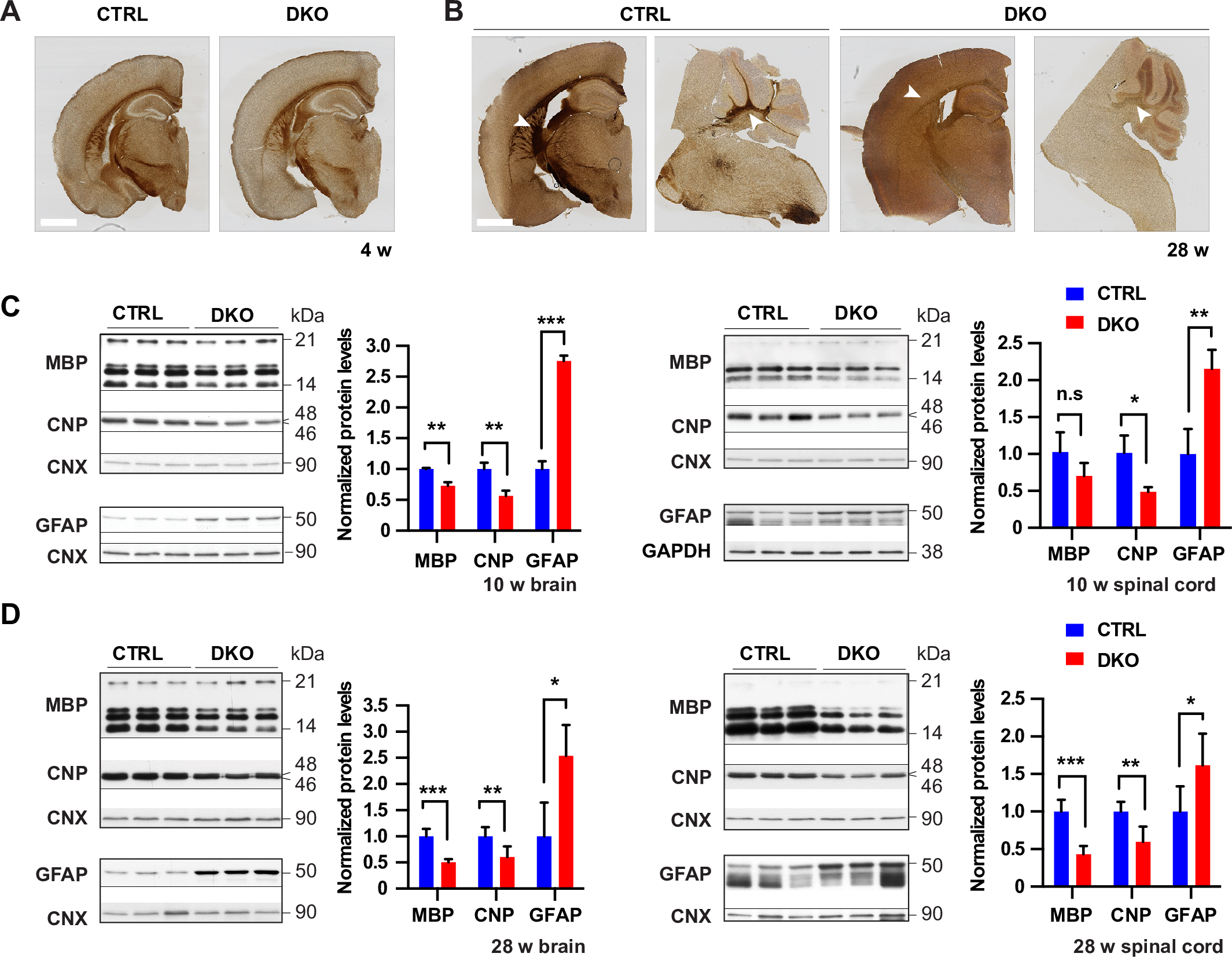

Supplement: S4 Fig — (A) Gallyas’ myelin staining of the forebrain at 4 weeks, before tamoxifen injection. Scale bar, 1 mm. (B) Gallyas’ myelin staining of the forebrain and cerebellum in 28-week-old mice. Arrowheads point to myelinated tracts. Scale bar, 1 mm. (C, D) Representative western blots and quantification of brain and spinal cord lysates of DKO mice at 10 weeks (10 w; n = 3 per genotype) and 28 weeks (28 w; n = 4–6 per genotype) of age. Student’s t-test, *p < 0.05, **p < 0.01, ***p < 0.001. Error bars are SD. (TIF) [file pgen.1006463.s004.tif]

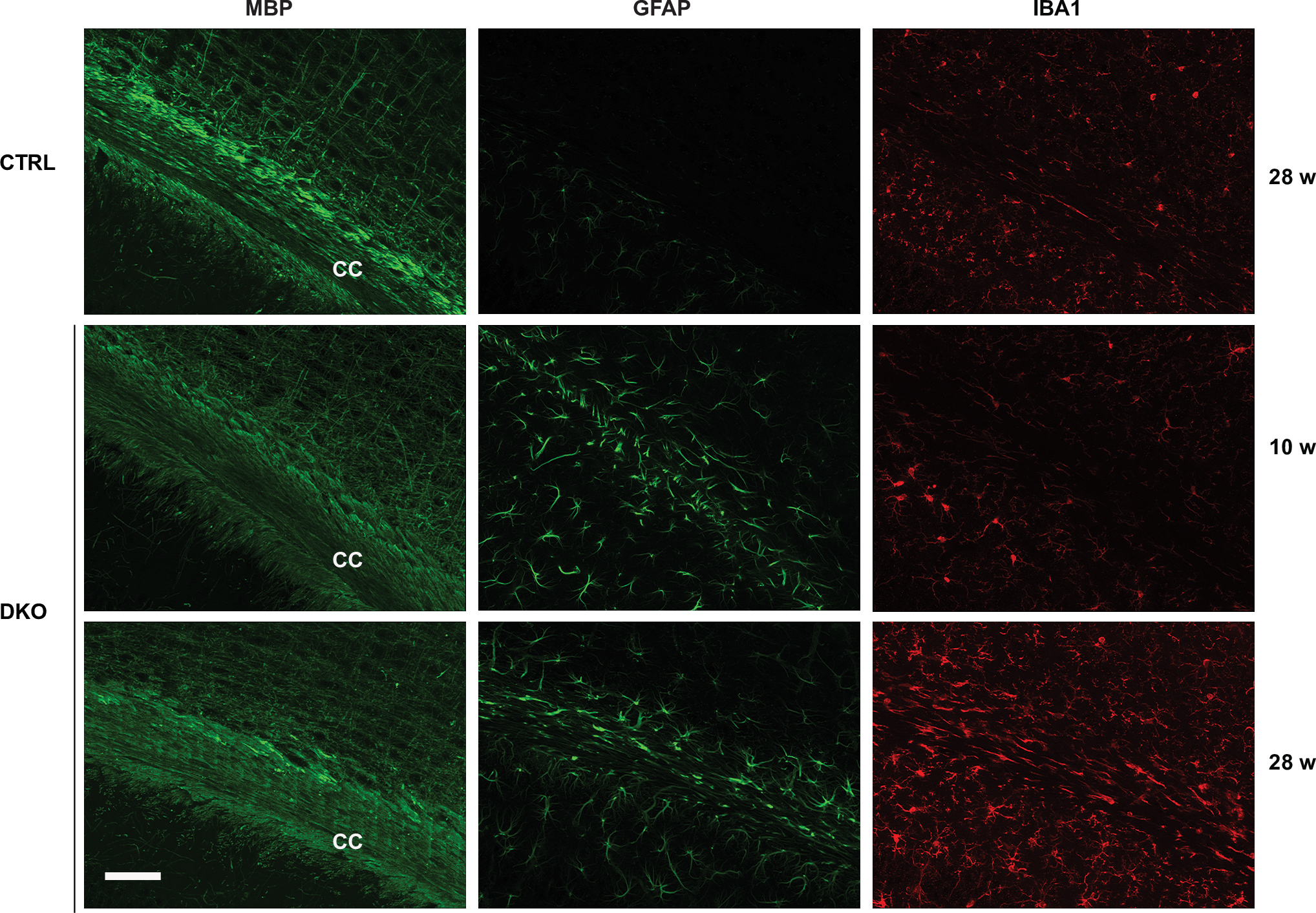

Supplement: S5 Fig — Immunofluorescence staining of MBP, GFAP, and IBA1 in the corpus callosum (CC) of CTRL and DKO mice at the indicated ages. Demyelination in the DKO mice is associated with astrocyte and microglia activation. Scale bar, 100 μm. (TIF) [file pgen.1006463.s005.tif]

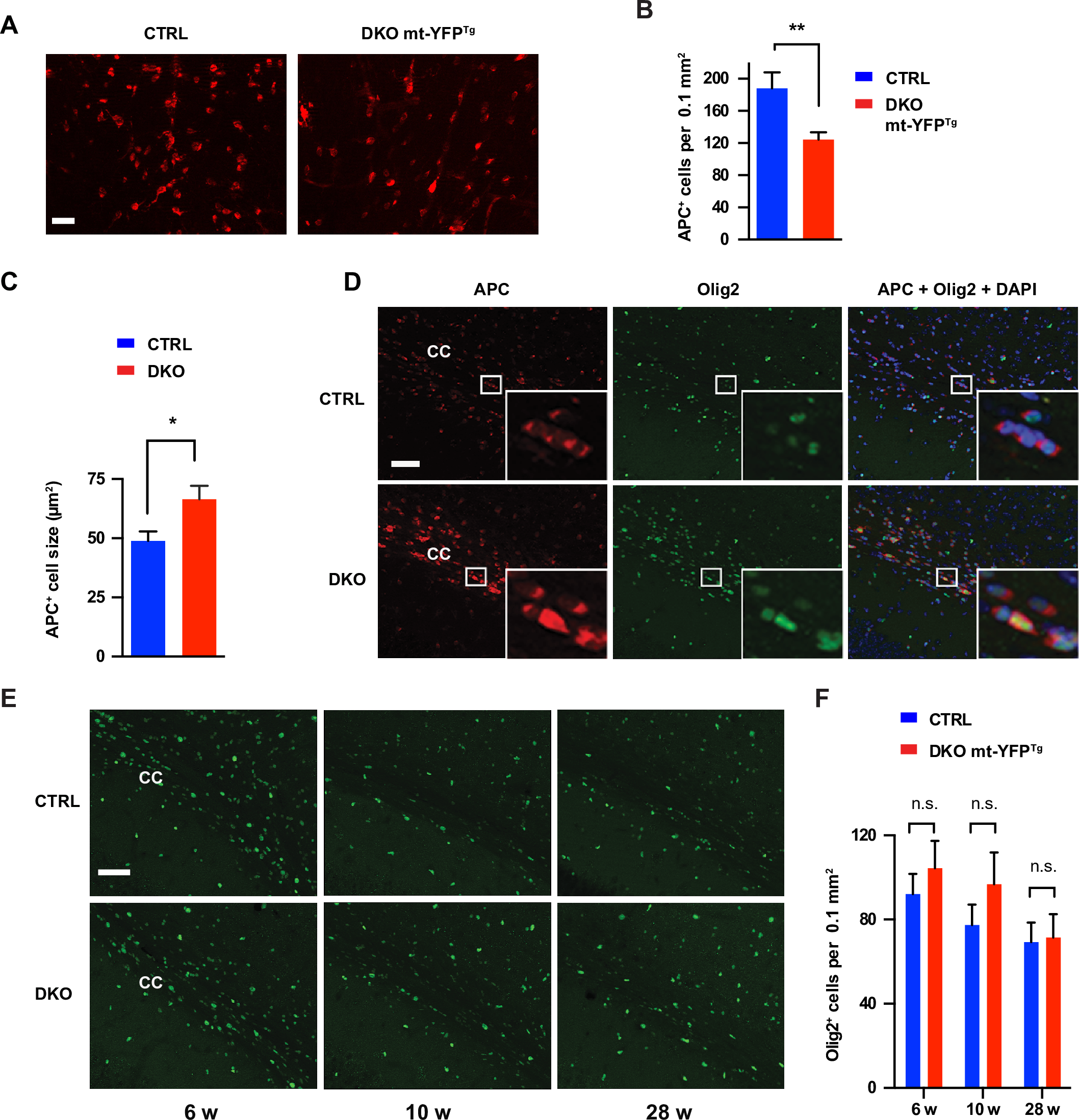

Supplement: S6 Fig — (A, B) Immunofluorescence staining and quantification of APC+ cells in the dorsal column of the spinal cord of 10-week-old CTRL and DKO mice. n = 3 mice per genotype. Student’s t-test, p < 0.01. Error bars are SD. (C) Quantification of the size of APC+ oligodendrocytes in the corpus callosum in 28-week-old mice. n = 3 mice per genotype (244 oligodendrocytes in the CTRL and 205 oligodendrocytes in the DKO were measured). Student’s t-test, p < 0.05. Error bars are SD. (D) Double immunofluorescence staining of APC and Olig2 in the corpus callosum of CTRL and DKO mice at 28 weeks. The enlarged APC+ oligodendrocytes in the DKO are more intensively stained by Olig2. Scale bar, 50 μm. (E) Immunofluorescence staining of Olig2+ cells in the corpus callosum (CC) of CTRL and DKO mice at the indicated age. n = 3 mice per genotype. (F) Quantification of Olig2+ cells in the corpus callosum (CC) of CTRL and DKO mice at the indicated age. n = 3 mice per genotype. Error bars are SD. (TIF) [file pgen.1006463.s006.tif]

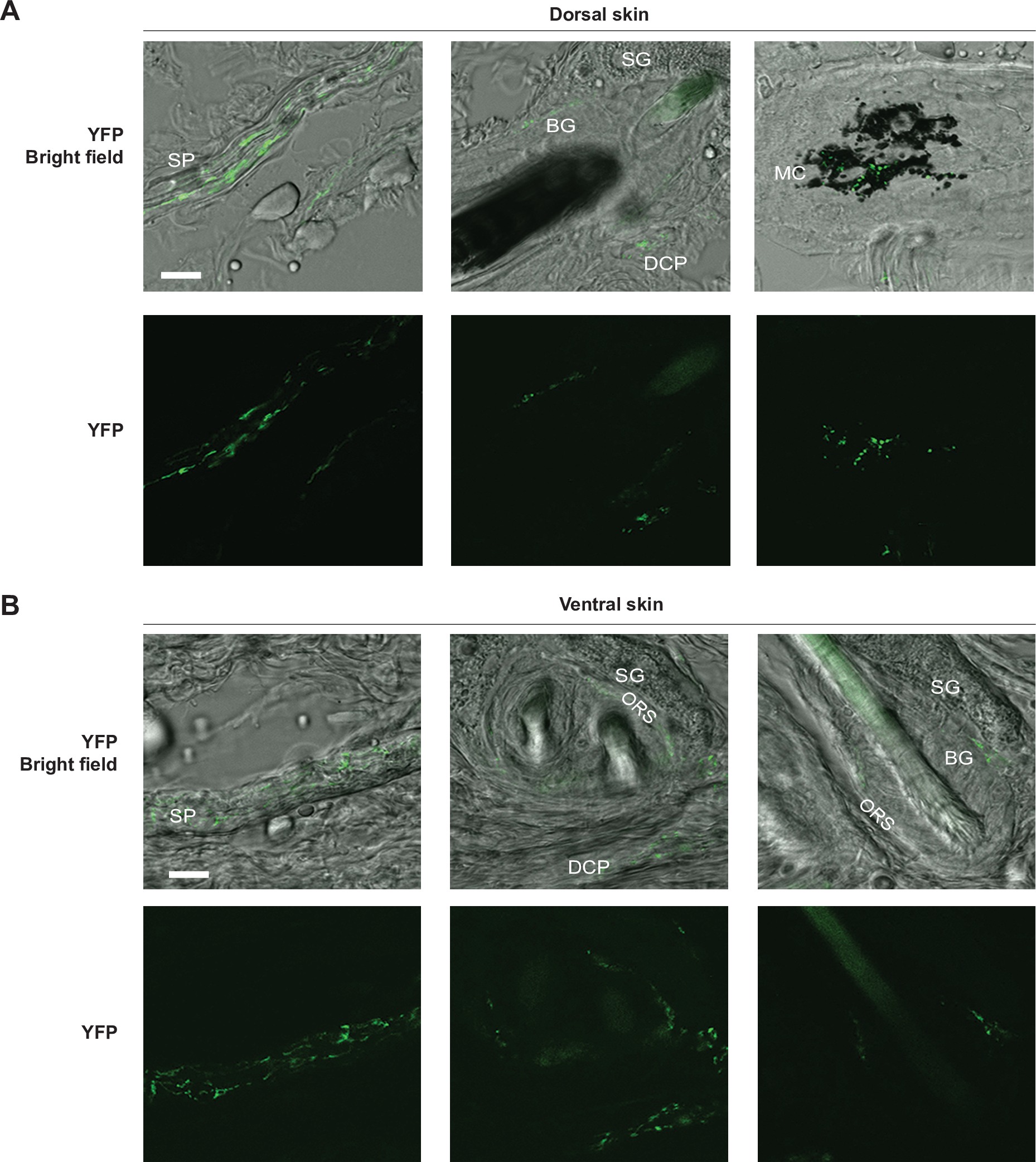

Supplement: S7 Fig — (A, B) Single-plane confocal images of dorsal (A) and ventral (close to forelimbs) (B) skin sections from Plp1-CreERT+/tg ROSA26+/SmY mice. Mice were injected with tamoxifen at P29 for five consecutive days and the skin was collected at P36. Endogenous mt-YFP+ signal in the cryosections is shown in green. SP: subcutaneous plexus; SG: sebaceous glands; BG: bulge area; DCP: deep cutaneous plexus; MC: melanocytes; ORS: outer root sheath. Scale bar, 10 μm. (TIF) [file pgen.1006463.s007.tif]
